# Genome Sequence of the Unusual Purple Photosynthetic Bacterium Phaeovibrio sulfidiphilus, Only Distantly Related to *Rhodospirillaceae*, Reveals Unique Genes for Respiratory Nitrate Reduction and Glycerol Metabolism

**DOI:** 10.1128/MRA.01200-20

**Published:** 2020-12-03

**Authors:** S. Dubey, T. E. Meyer, J. A. Kyndt

**Affiliations:** a College of Science and Technology, Bellevue University, Bellevue, Nebraska, USA; b Department of Chemistry and Biochemistry, the University of Arizona, Tucson, Arizona, USA; Georgia Institute of Technology

## Abstract

Phaeovibrio sulfidiphilus was reported to be a divergent member of the purple photosynthetic bacteria with limited ability to metabolize organic compounds. Whole-genome-based analysis shows that it is indeed only distantly related to freshwater species of *Rhodospirillaceae*. Unexpectedly, the genome contains unique gene clusters for potential respiratory nitrate reduction and anaerobic glycerol metabolism.

## Abstract (precis)

Phaeovibrio sulfidiphilus was reported to be a divergent member of the purple photosynthetic bacteria with limited ability to metabolize organic compounds. Whole-genome-based analysis shows it is indeed only distantly related to freshwater species of *Rhodospirillaceae*. Unexpectedly, the genome contains unique gene clusters for potential respiratory nitrate reduction and anaerobic glycerol metabolism.

## ANNOUNCEMENT

*Phaeovibrio sulfidiphilus* is unusual in having both lamellar membrane stacks and vesicular membranes, which so far is unique (1). It has limited ability to utilize organic compounds for photosynthetic growth, consisting of acetate, pyruvate, and succinate. It is an obligate anaerobe and has a requirement for sulfide as a sulfur source, although apparently not using it as an electron donor (1).

Phaeovibrio sulfidiphilus DSM23193 was originally isolated from brackish water near Nagapattinam, India (1). Cells were grown and genomic DNA was prepared by the DSMZ (Deutsche Sammlung von Mikroorganismen und Zellkulturen, GmbH) culture collection. DNA was purified using the MasterPure Gram-positive DNA purification kit (Lucigen). Qubit and NanoDrop DNA analysis showed an absorbance 260/280 ratio of 1.81. The sequencing library was prepared using the Illumina Nextera DNA Flex library prep kit. The genome was sequenced with an Illumina MiniSeq instrument using 500 μl of a 1.8 pM library. Paired-end (2 × 150 bp) sequencing generated 3,390,406 reads and 257.97 Mbp (100× coverage). Quality control of the reads was performed using FASTQC within BaseSpace version 1.0.0 (Illumina), using a k-mer size of 5 and contamination filtering. We assembled the genome *de novo* using Unicycler version 0.4.8 through PATRIC (2, 3). This assembly yielded 31 contigs (>300 bp), the largest being 750,242 bp, and an *N*_50_ value of 300,711 bp. The genome was 2,585,463 bp long with a GC content of 66.96%. The genome was annotated using RASTtk version 1.3.0 (4) within PATRIC (2). This showed our strain to have 2,214 coding sequences and 50 tRNAs. An EvalG genome quality analysis, using the CheckM algorithm (5), ran during PATRIC annotation and showed an estimated 100% completeness and 0% contamination for the Phaeovibrio sulfidiphilus genome. Default parameters were used for all software unless otherwise noted.

**FIG 1:**
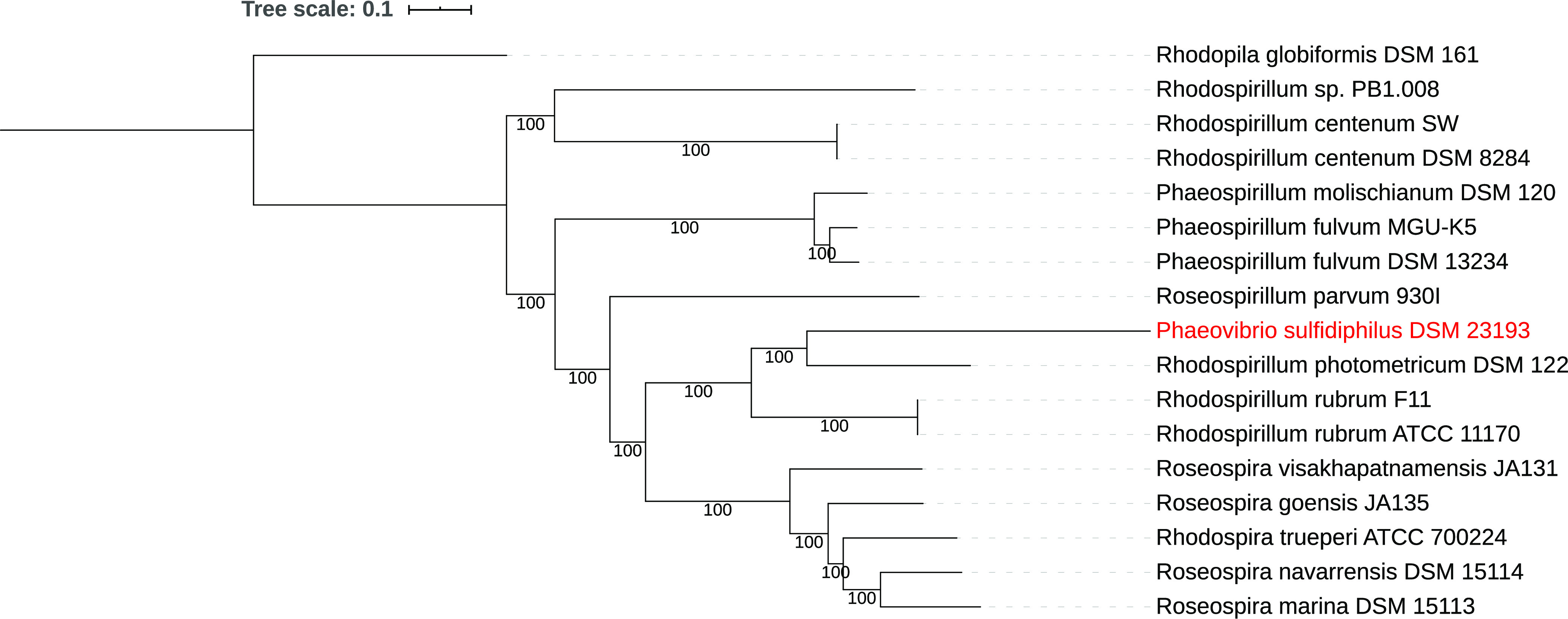
Whole-genome-based phylogenetic tree of Phaeovibrio sulfidiphilus and its closest relatives. The phylogenetic tree was generated using the CodonTree method within PATRIC (2), which used cross-genus families (PGFams) as homology groups. A total of 316 PGFams were found among these selected genomes using the CodonTree analysis, and the aligned proteins and coding DNA from single-copy genes were used for RAxML analysis (5, 6). The support values for the phylogenetic tree were generated using 100 rounds of the “rapid bootstrapping” option of RAxML (2). Rhodopila globiformis DSM 161 was used as an outgroup (12). Interactive Tree of Life (iTOL) was used for the tree visualization (13).

According to 16S rRNA analysis, *P. sulfidiphilus* is a distant relative of the freshwater *Rhodospirillaceae*, such as Rhodospirillaceae rubrum, Pararhodospirillum photometricum, and Pararhodospirillum oryzae (1). A JSpecies comparison (6) of the average nucleotide identity (ANI) showed only 71% to *R. rubrum*, *P. photometricum*, and *P. oryzae*, which is near the limit of usefulness of this technique (6). We performed a whole-genome phylogenetic analysis of the Phaeovibrio sulfidiphilus genome using RAxML within PATRIC (7, 8) (Fig. 1), which shows that this species is clearly different from the *Rhodospirillum*, *Rhodospira*, and *Roseospira* genera and rightfully belongs in its own genus (Fig. 1).

*P. sulfidiphilus* was found to be strictly anaerobic (1). This can be understood from the genome sequence in that most photosynthetic species produce the high-affinity FixNOP oxidase, which is generally known for protection of nitrogenase but is also shown to protect against the harmful effects of oxygen on the species as a whole (9). *P. sulfidiphilus* does not have nitrogenase and does not have the FixNOP oxidase either, explaining their strict anaerobic growth requirement. *P. sulfidiphilus* has a large cytochrome *c*_2_ like *R. rubrum* has (10) but also has the membrane-bound PufC tetraheme cytochrome *c* like *Pararhodospirillum* species have (11). The genome contains a unique gene cluster with respiratory nitrate reductase subunit genes (*narGHIJ*) and two nitrate/nitrite transporters (Table 1) not found in any of the close relatives. It also contains a unique gene family cluster for anaerobic glycerol-3-phosphate dehydrogenase (*glpABC*) genetically linked to a glycerol uptake facilitator protein and a glycerol kinase (Table 1). Based on these unique gene clusters, it is expected that *P. sulfidiphilus* would be able to grow anaerobically on glycerol and use nitrate as an electron acceptor, which is in contrast to the earlier growth studies (1), warranting further physiological studies.

**TABLE 1:** Unique protein families identified in Phaeovibrio sulfidiphilus that are not present in any of the related *Rhodospirillaceae* species in Fig. 1^*a*^

Protein family ID^*b*^	Description
PGF_00055324	Anaerobic glycerol-3-phosphate dehydrogenase subunit A (EC 1.1.5.3)
PGF_03080445	Anaerobic glycerol-3-phosphate dehydrogenase subunit B (EC 1.1.5.3)
PGF_07625325	Anaerobic glycerol-3-phosphate dehydrogenase subunit C (EC 1.1.5.3)
NA	Glycerol uptake facilitator protein
PGF_00008537	Glycerol kinase (EC 2.7.1.30)
PGF_10329010	Fe-S oxidoreductase
PGF_04457374	Nitrate/nitrite transporter NarK/U
PGF_10490354	Nitrate/nitrite transporter NarK/U 1/nitrate/nitrite transporter NarK/U
PGF_09372748	Respiratory nitrate reductase alpha chain (EC 1.7.99.4)
PGF_00047730	Respiratory nitrate reductase beta chain (EC 1.7.99.4)
NA	Respiratory nitrate reductase delta chain (EC 1.7.99.4)
NA	Respiratory nitrate reductase gamma chain (EC 1.7.99.4)
PGF_05493644	Sulfur carrier protein ThiS adenylyltransferase (EC 2.7.7.73)

a Respiratory nitrate reductase genes were found clustered with two nitrate/nitrite transporters, and the anaerobic glycerol metabolism gene cluster contains a glycerol-3-phosphate dehydrogenase (subunits A, B, and C), glycerol uptake facilitator protein, and glycerol kinase.

b NA, no PGFam ID was assigned in PATRIC to the annotated genes.

### Data availability.

This whole-genome shotgun project has been deposited at DDBJ/ENA/GenBank under the accession number JACZHT000000000. The version described in this paper is version JACZHT010000000. The raw sequencing reads have been submitted to the SRA, and the corresponding accession number is SRR12806899.
